# Baseline Eosinophil Count as a Potential Clinical Biomarker for Clinical Complexity in EGPA: A Real-Life Experience

**DOI:** 10.3390/biomedicines10112688

**Published:** 2022-10-24

**Authors:** Andrea Matucci, Emanuele Vivarelli, Margherita Perlato, Valentina Mecheri, Matteo Accinno, Lorenzo Cosmi, Paola Parronchi, Oliviero Rossi, Alessandra Vultaggio

**Affiliations:** 1 Immunoallergology Unit, Careggi University Hospital, 50134 Florence, Italy; 2 Department of Clinical and Experimental Medicine, Unit of Internal Medicine, University of Florence, 50121 Florence, Italy

**Keywords:** eosinophilic granulomatosis with polyangiitis, eosinophils, biomarker, asthma, nasal polyps, peripheral neuropathy

## Abstract

Background: Eosinophilic granulomatosis with polyangiitis (EGPA) is a small-vessel necrotizing vasculitis with multiple organ involvement. Despite improvements in clinical management, biomarkers for organ involvement and disease prognosis are still an unmet need. Methods: EGPA patients referred to our immunology clinic were retrospectively reviewed. Demographic/clinical features, eosinophils, ANCA status, eosinophil cationic protein (ECP) and total serum IgE were evaluated at the baseline. Eosinophils, total serum IgE, ECP and ANCA were studied as possible biomarkers for lung and extrapulmonary disease. Results: In total, 167 EGPA patients were recruited for our study. A positive association between eosinophils and peripheral nervous system (PNS) involvement was demonstrated (*p* <0.001; chi-squared test). Receiver operating characteristic (ROC) curves using the eosinophil count or percentage as predictors of PNS involvement yielded AUC values of 0.75 and 0.67, respectively. ANCA positivity was associated with PNS involvement, while no correlations with clinical parameters were found for ECP and total serum IgE. Patients without extrapulmonary involvement had lower eosinophils (eosinophils: 2844.7 ± 1698 vs. 6373 ± 5468, *p* < 0.001; eosinophil percentage: 24.6 ± 10% vs. 36.2 ± 15.8, *p* < 0.001) and were less likely to be ANCA+ (*p* < 0.001, chi-squared test). Conclusion: Eosinophils in EGPA are an important biomarker and are associated with extrapulmonary involvement. These findings could strengthen the role of anti-eosinophilic drugs in improving extrapulmonary disease.

## 1. Introduction

Eosinophilic granulomatosis with polyangiitis (EGPA) is a small-vessel necrotizing vasculitis with multiple organ involvement, with the lung being the most commonly affected [1,2]. A direct pathogenic effect of eosinophils infiltrating different tissues is considered one of the main drivers of EGPA pathophysiology, although the pathogenesis of EGPA is not fully defined [3,4].

In the American College of Rheumatology (ACR) criteria, EGPA is defined by the presence or a history of asthma, a blood eosinophil percentage higher than 10% or an absolute count >1.000 cells/mm^3^ and the presence of at least two of the following features: (1) biopsy showing histopathological evidence of eosinophilic vasculitis, perivascular eosinophilic infiltration or eosinophil-rich granulomatous inflammation; (2) pulmonary infiltrates, non-fixed; (3) sino-nasal abnormalities as chronic rhinosinusitis with nasal polyps (CRSwNP); (4) neuropathy, characterized by mono- or poly-motor deficit or nerve conduction abnormality [5]. Recently, the ACR/European Alliance of Associations for Rheumatology has proposed new classification criteria for EGPA. These criteria confirm the importance of blood eosinophilia and asthma in distinguishing EGPA from other forms of vasculitis [6].

Eosinophils are the prevailing innate immunity cells involved in EGPA inflammation, meaning blood and tissue eosinophilia are disease pathognomonic findings. EGPA is usually regarded as a Th2-driven inflammatory disease; autoimmunity can also develop, as can be seen by the anti-neutrophil cytoplasmic antibodies’ (ANCAs’) production [7]. The effector function of eosinophils is related to the release of many mediators capable of inducing tissue injury, but also of cytokines and chemokines that amplify the inflammatory process [8]. IL-5, mainly produced by T-cells but also by IL-3 and granulocyte macrophage colony stimulating factor (GM-CSF), can prime eosinophils and prompt them express all of the membrane receptors and integrins mandatory for tissue migration [9]. Tissue migration is a complex process mainly driven by chemokines, such as eotaxins-3 (CCL26), RANTES (regulated on activation of normal T cells, expressed and secreted; CCL5), monocyte chemoattractant protein (MCP)-3 (CCL7) and MCP-4 (CCL13) [10,11,12]. The existence of cross-talk between eosinophils and T cells in the inflammatory process is further demonstrated by the production of IL-25 by eosinophils. In fact, this alarmin induces the switch of T cells toward a Th2-phenotype and the activation of type 2 innate lymphoid cells (ILC2), which are significantly increased in EGPA patients and produce high amounts of IL-5 [13,14,15].

The complexity of the EGPA pathogenic mechanisms is highlighted by the demonstration of high levels of circulating Th17 cells [16]. Moreover, in patients with active or relapsing EGPA, T regulatory (Treg) cells counts were low and increased percentages of B lymphocytes were found [17].

The EGPA pathogenesis reveals itself in all its complexity when we consider the presence of granulomas, often associated with necrosis, which are considered a histological marker of the disease. In fact, they do not only contain eosinophils but also macrophages and neutrophils as the result of both Th2 and Th1/Th17 responses [18].

Asthma represents a hallmark of EGPA since it is present in almost all patients, as shown by epidemiological data.

In EGPA patients, clinical manifestations vary according to the phase of the disease. Patients usually suffer from asthma and nasal polyposis for several years before developing hypereosinophilia and organ infiltration. Furthermore, the involvement of different areas of the body leads to different clinical phenotypes. The upper airways, peripheral nervous system, kidneys (especially in ANCA+ subjects), myocardium (especially in ANCA- patients), lungs, GI tract and skin are the most common targets of the disease [19,20,21]

As previously stated, the existence of an autoimmune response in EGPA patients has been confirmed by the detection of ANCA in the sera of about 40% of patients [19]. However, it has been shown that sputum ANCA positivity is detectable in more than 70% of EGPA patients, irrespective of their serum ANCA status [20,21,22,23,24]. Treatment mainly relies on systemic corticosteroids and immunosuppressive agents [20,25]. The central role of IL-5 in the pathogenesis of EGPA has paved the way for the use of the anti-IL-5 monoclonal antibody (mAb) mepolizumab. The current evidence on the efficacy and safety of mepolizumab in EGPA has led to the approval of its use for the treatment of these patients [26,27].

In this paper, we retrospectively review the clinical and laboratory features of our EGPA cohort, with the aim to identify biomarkers potentially useful for clinical practice.

## 2. Materials and Methods

### 2.1. Study Population

**Figure 1:**
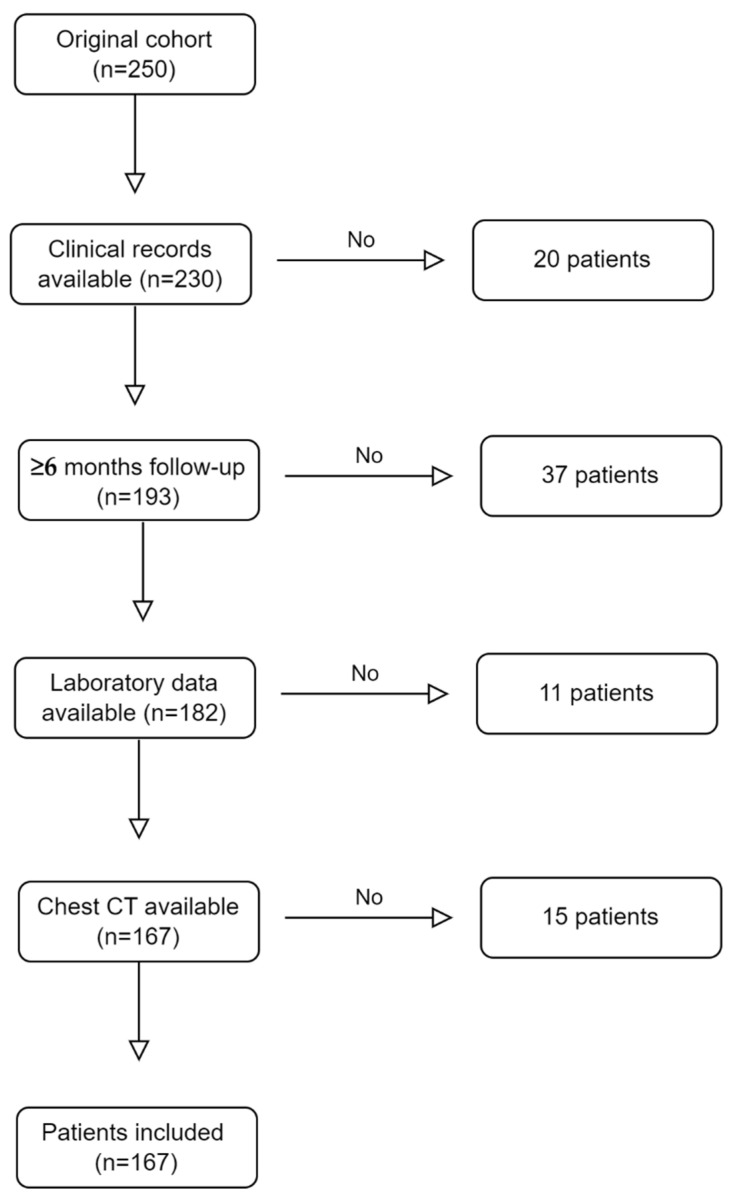
Inclusion and exclusion criteria for the study enrollment. CT: chest tomography.

Among patients referred to our immunology clinic from January 2010 to December 2021, patients fulfilling the 1990 ARA EGPA classification criteria were enrolled (Figure 1). Demographic, clinical and laboratory data were retrieved from their clinical records. The blood eosinophil count (BEC), total IgE (CAP-FEIA, ThermoFisher, Uppsala, Sweden), atopic status, eosinophil cationic protein (ECP, CAP-FEIA, ThermoFisher, Uppsala, Sweden) and ANCA (EUROPLUS Granulocyte Mosaic 25, IFA, Euroimmun, Lubeck, Germany; EliaTM MPOS, EliaTM PR3S, ThermoFisher, Uppsala, Sweden) were analyzed at the baseline. Atopy was defined as skin test positivity and/or documented serum-specific IgE for inhalant allergens. All patients underwent nasal endoscopy and a head computed tomography (CT) scan to investigate sino-nasal involvement at the baseline. Lung involvement was assessed with a chest CT scan at the baseline in all patients. All patients were screened for heart involvement by transthoracic echocardiography; more advanced heart assessments, such as cardiac magnetic resonance (MRI) or coronary angiography, were performed according to the patients’ clinical needs. All patients were screened for kidney involvement using laboratory (serum creatinine and urinalysis) and ultrasound assessment. Other clinical actions were physician-assessed.

### 2.2. Blood Eosinophil Grouping Strategy

To analyze possible correlations between BEC and clinical features, we divided the patients in two groups: (i) “extremely high eosinophils” group (EH-Eo group), in which blood eosinophils were >10.000/μL; (ii) “high eosinophils” group (H-Eo group), with blood eosinophils ≤10.000/μL). To better analyze the correlations of eosinophil blood levels with the clinical outcomes, we divided the HEo group according to quartiles. We, therefore, obtained five groups: first group (740–1960 cells/microL; 37 subjects); second group (1980–3500 cells/microL; 38 subjects); third group (3590–5422 cells/microL; 41 subjects); fourth group (5520–9830 cells/microL; 32 subjects); fifth group (10,580–32,614 cells/microL; 19 subjects).

### 2.3. Statistical Methodology

Statistical analysis was performed using Python version 3.8.0 (Anaconda distribution, Open source software). Spearman’s correlation, the X-squared test, Mann–Whitney U test and survival analysis were used when appropriate. *p*-values lower than 0.05 were considered statistically significant.

## 3. Results

### 3.1. Patients’ Characteristics

We recruited a cohort of 167 EGPA patients with a prevalence of the male sex (100/167; 59.9%) and an overall mean age at diagnosis of 49.9 ± 12.9 years, while the mean age of symptom onset was 46.3 ± 13.6 years. These data imply a significant diagnostic delay, which was calculated as 1.6 ± 2.3 years (five patients with diagnostic delays of over five years were considered outliers and excluded from this calculation). The age distribution at diagnosis was consistent with the known medical literature, with the majority of patients aged from 28 to 72 years; no EGPA patients younger than 17 or over 83 years were found in our cohort. ANCA (exclusively anti-myeloperoxidase antibodies) were present in 38.3% of the patients (64/167), while the mean blood eosinophils count was 5422 ± 5002 cells/μL. Atopy was demonstrated in 67 out of 167 (40%) of our cohort.

**Figure 2:**
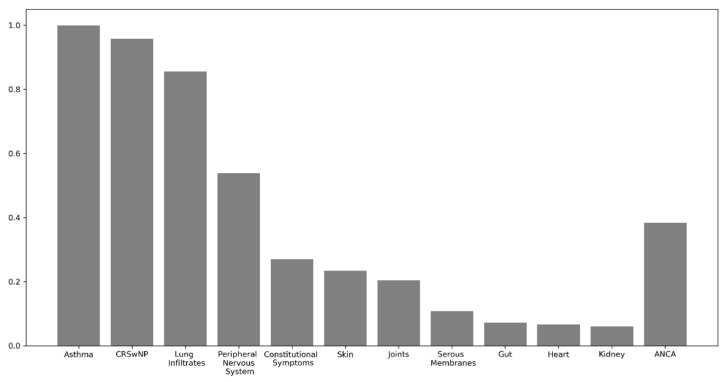
Detailed organ involvement in our cohort. CRSwNP: chronic rhinosinusitis with nasal polyps.

Concerning the clinical involvement at the baseline, all patients were long-standing asthmatics (asthma duration at diagnosis 9.9 ± 10.4 years), with a severe functional impairment as shown by a mean baseline FEV1 of 69.5% ± 22. Furthermore, most of them showed, in their clinical history, alveolar opacities (144/167; 86.2%). Other common clinical features were CRSwNP (157/167; 94%) and peripheral nervous system (PNS) involvement including mononeuritis multiplex and polineuropathy (90/167; 53.9%). A significant number of patients experienced, in their clinical history, skin rashes including urticaria and skin vasculitis (41/167; 24.5%) and arthralgias/arthritis (36/167; 21.6%). Only a minority of patients displayed clinical or laboratory signs of serositis, kidney, heart or central nervous system involvement. All relevant data concerning our study population are summarized in Table 1. The clinical involvement is summarized in Figure 2.

### 3.2. Eosinophils Are Associated with PNS Involvement

**Figure 3:**
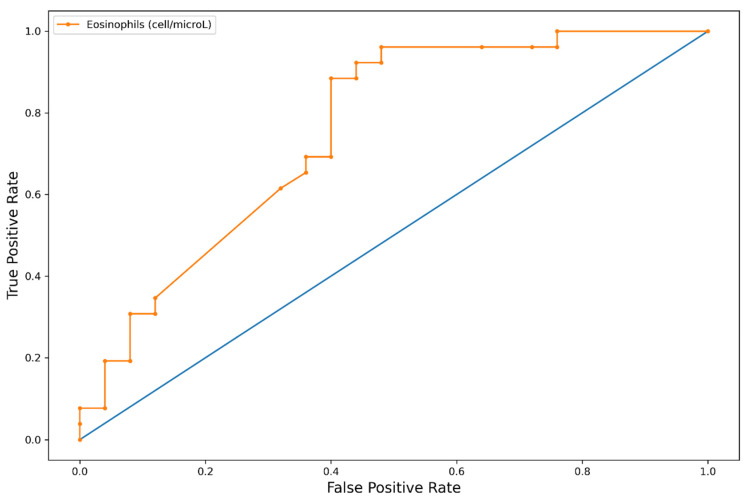
ROC curves with PNS involvement as the dependent variable and the eosinophil count as the independent variable.

**Figure 4:**
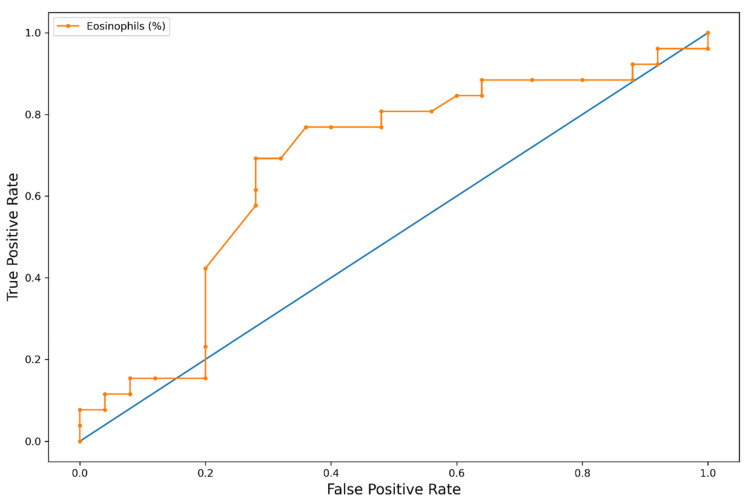
ROC curves with PNS involvement as the dependent variable and the eosinophil percentage as the independent variable.

When we evaluated the clinical manifestations of EGPA according to the five groups of blood eosinophils, we observed a positive association between eosinophil counts and the percentages of PNS involvement (*p* < 0.001; chi-squared test). A receiver operating characteristic (ROC) curve using the absolute eosinophil count as the independent variable and PNS involvement as the dependent variable yielded an AUC value of 0.75, while the same ROC curve using the eosinophil percentage yielded an AUC value of 0.67 (Figure 3 and Figure 4). No patients with eosinophil counts lower than 1150/μL showed PNS involvement. We also evaluated the eosinophil blood count as a biomarker for PNS involvement, using the literature cutoff for hypereosinophilic syndrome (blood eosinophils >1500 cells/microL). According to this cutoff value, the test sensitivity was high (94.4%), but its specificity was rather low (26%). The classical eosinophil cutoff percentage for EGPA diagnosis (10%) could not be evaluated due to the low number of patients showing baseline blood eosinophils lower than 10%.

**Figure 5:**
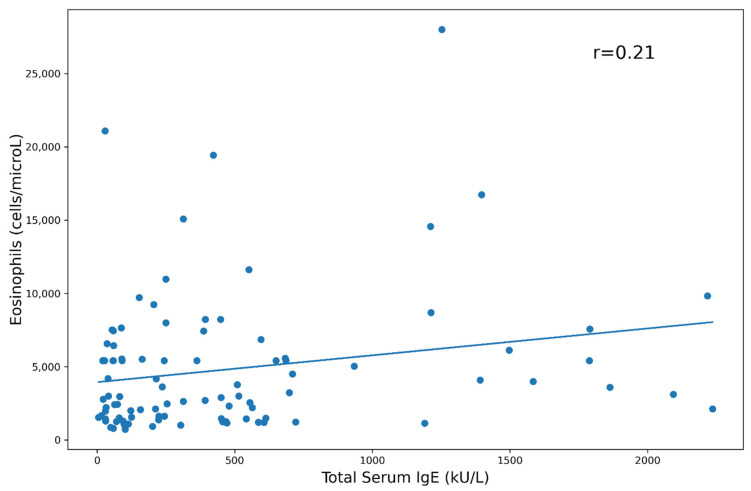
Eosinophils absolute count vs. total IgE.

**Figure 6:**
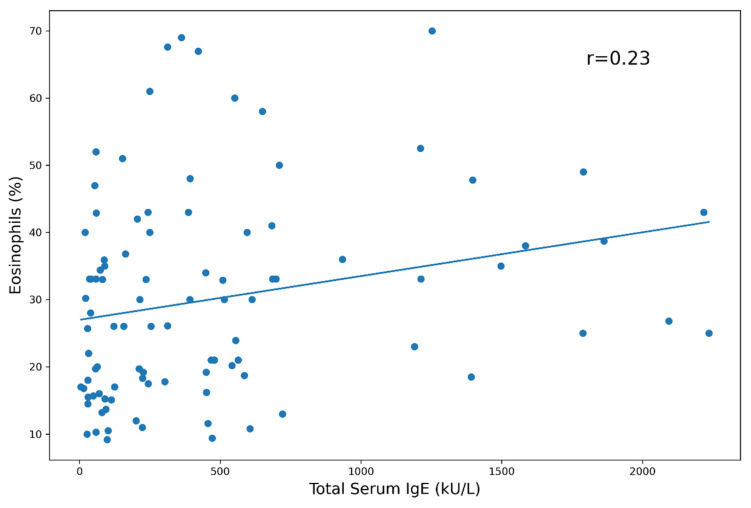
Eosinophils percentage vs. total IgE.

The blood eosinophil count was not associated with atopy, but it displayed a positive correlation with the total serum IgE levels (Pearson’s correlations = 0.21 and 0.23, *p* < 0.05; Figure 5 and Figure 6). Furthermore, the blood eosinophil count did not show any association or correlation with demographic features, ANCA status or other organ involvement. ECP did not correlate with any clinical, laboratory or demographic parameter.

### 3.3. ANCA Are Associated with PNS Involvement

To further characterize the relationship between neural nervous system involvement and other markers, we observed that ANCA+ patients showed more PNS symptoms when compared to ANCA- patients (49/64 (76.6%) vs. 41/103 (39.8%), *p* < 0.001, chi-squared test). There was no difference in ANCA status when we stratified patients for sex, age at diagnosis, eosinophils, ECP, atopy or other organ involvement.

### 3.4. EGPA Phenotypes: The “Only-Lung EGPA” Patients

In our cohort, we could identify two patient phenotypes. First, 45 out of the 167 (26.9%) patients showed asthma, eosinophilia and CRSwNP with lung opacities as the only organ involvement; we called this group “only-lung EGPA”. The remaining patients (122/167; 73.1%), displaying the involvement of additional organs, were called the “lung-plus EGPA” group. When comparing the two groups, we observed that “only-lung EGPA” patients had significantly lower eosinophil levels than the “lung-plus EGPA” patients (eosinophil count: 2844.7 ± 1698 vs. 6373 ± 5468, *p* < 0.001; eosinophil percentage: 24.6 ± 10% vs. 36.2 ± 15.8, *p* < 0.001) and were less likely to be ANCA+ (7/45 (15.6%) vs. 57/122 (46.7%), *p* < 0.001, chi-squared test). There were no significant differences between the two groups when looking at gender, atopy or age at diagnosis.

## 4. Discussion

Our case series allows us to confirm the importance of asthma and CRSwNP in EGPA patients. In fact, we not only observed that virtually all patients had both asthma and CRSwNP but also that these conditions preceded the full-blown disease by a few years [7]. Although the pathogenesis of EGPA is a complex scenario, it is very likely that asthma and EGPA share common pathogenic aspects. It is well-known that asthma is frequently characterized by an increase in blood eosinophils that as well as releasing several mediators and cytokines, are also involved in tissue damage [28,29,30]. Therefore, over time, severe forms of eosinophilic asthma can evolve in EGPA. Although a specific cut-off of blood eosinophils has not been defined, patients with “high/very high” blood eosinophil levels should be carefully evaluated to distinguish those with isolated severe eosinophilic asthma from those with asthma in EGPA disease [31]. Clinicians must consider possible “red flags” for the risk of EGPA development, such as a history of persistent asthma, more often non-allergic and late-onset, the presence of nasal polyposis, the presence or history of hypereosinophilia and aspirin intolerance. Clinicians should always be aware that patients with severe asthma who are oral corticosteroids (OCS)-dependent can have underlying ANCA-negative EGPA. In these cases, a close follow-up should be implemented. The underestimation of these simple clinical data explains the delay in the diagnosis of EGPA, as confirmed in our cohort. Moreover, diagnostic delay may be responsible for the onset of severe EGPA features with extra-pulmonary involvement including, in our experience, peripheral neuropathy. It is interesting to observe the direct association between the high number of eosinophils and NPS involvement. Of note, in our case series, patients with the lowest eosinophils count (about 1000 cells/mm^3^ or less) showed neither PNS involvement nor other extra-pulmonary involvement, as well as a lower ANCA-positivity rate. To our knowledge, eosinophils are an item of EGPA classification criteria but not a potential biomarker for multiorgan involvement.

The clinical significance of the association of eosinophils with extrapulmonary involvement, such as PNS, was confirmed by the AUC value of the ROC curve observed in our cohort. This finding supports the pathogenetic role of these cells in EGPA, particularly in inducing organ damage. It was shown that damage to the vascular structures of the nerves (disruption of vascular layers and/or obstruction of the lumen) occurred more frequently in specimens from ANCA+ patients than those from the ANCA- group. In addition, fibrinoid necrosis was observed in 40% and 5% of epineurial vessels in the ANCA+ and ANCA- groups, respectively [32].

In ANCA- patients, the damage to nervous fibers is due to the accumulation of eosinophils in the endoneurium, as they release eosinophil-derived neurotoxin (EDN), major basic protein (MBP), eosinophil peroxidase and eosinophil cationic protein (ECP) stored in their cytoplasmatic granules [9,32]. In addition, eosinophils cause the occlusion of the lumen of epineurial vessels, inducing ischemic damage. It is important to note, at least in our case series, that there is no association between the peripheral blood eosinophil level, as a percentage or absolute value, and ANCA-positivity.

Considering the role of eosinophils in organ injury, it is possible to suppose that treatment with anti-IL-5/IL-5R mAbs might allow us to improve, or prevent, not only lung disease but also extra-pulmonary involvement.

Our patients showed high serum levels of total IgE, though a specific sensitization toward inhalant allergens was demonstrated only in a small percentage, and a positive correlation between IgE levels and eosinophil counts. This can be explained by the production of IL-4 and IL-13 by Th2 lymphocytes in addition to IL-5, further underlying the role of this cell subset in EGPA pathogenesis; however, it should be emphasized that high IgE serum levels are demonstrated in many allergic disease and are not a specific feature of EGPA [14]. It was also observed that ILC2 produces not only IL-5 but also IL-4 and IL-13, thus further stimulating IgE production in eosinophilic asthma and EGPA [15,33].

In conclusion, our study confirms not only that asthma and CRSwNP are hallmarks of EGPA but also that they predate the full-blown disease by several years. Moreover, eosinophils are certainly important in the pathogenesis of organ injury, and their absolute count is a “biomarker” associated with extra-pulmonary involvement.

## Figures and Tables

**Table 1:** Baseline demographic, clinical and laboratory characteristics of the study population. Data are presented as the mean ± standard deviation or number (%).

Demographic, Clinical and Laboratory Features	All Patients (*n* = 167)
Female	100/167 (60)
Age at diagnosis (y)	49.93 ± 12.92
Age at symptoms onset (y)	46.27 ± 13.6
Diagnostic delay (y)	1.61 ± 2.32
Atopy	67/167 (40)
Asthma	167/167 (100)
Lung opacities	144/167 (86.2)
CRSwNP	157/167 (94)
PNS involvement	90/167 (53.9)
Serositis	18/167 (10.8)
Skin involvement	41/165 (24.8)
Arthralgia/arthritis	36/165 (21.6)
Heart involvement	10/167 (6)
Glomerulonephritis	10/167 (6)
CNS involvement	1/167 (0.5)
Constitutional symptoms	43/167 (25.7)
Baseline FEV1	69.52% ± 21.98
Blood eosinophils (cells/μL)	5422 ± 5002
Blood eosinophils (%)	33.1 ± 15.4
ECP (μg/L)	75.7 ± 98.2
Total IgE (kU/L)	509.4 ± 669.4
ANCA+	64/167 (38.3)

ANCA: anti-neutrophil cytoplasmic antibodies; CNS: central nervous system;. CRSwNP: chronic rhinosinusitis with nasal polyps; ECP: eosinophil cationic protein; FEV1: forced expiratory volume in the first second; PNS: peripheral nervous system.

## Data Availability

Data are available on reasonable request from E.V.
